# Spore-Forming Probiotics and Their Mechanisms of Action: A Particular Focus on *Alkalihalobacillus clausii*

**DOI:** 10.3390/nu18091378

**Published:** 2026-04-27

**Authors:** Diletta Mazzantini, Emilia Ghelardi

**Affiliations:** 1 Department of Translational Research and New Technologies in Medicine and Surgery, University of Pisa, 56123 Pisa, Italy; diletta.mazzantini@unipi.it; 2 Research Center Nutraceuticals and Food for Health—Nutrafood, University of Pisa, 56123 Pisa, Italy

**Keywords:** gut microbiota, probiotics, spore-formers, human diseases

## Abstract

Gut microbiota play crucial roles in host health, including immune regulation, metabolism, and nutrient absorption. Its dysregulation has been linked to various diseases. When administered in sufficient amounts, probiotics can contribute to restoring the gut microbial balance and maintain homeostasis. Species of the genus *Bacillus* and related genera (*Alkalihalobacillus* and *Heyndrickxia*) have been extensively studied and commercialized as probiotics due to their ability to form endospores, the dormant cell forms that provide remarkable resistance to adverse conditions. Understanding the mechanisms of the action of spore-forming probiotics is essential for harnessing their therapeutic potential. This review explores the mechanisms underlying the action of spore-forming probiotics, with a special focus on *Alkalihalobacillus clausii*. Many beneficial properties such as resilience in extreme conditions, multiplication in the gut, gut adhesion, immunomodulatory effects, the production of bioactive and antimicrobial compounds, as well as efficacy in human health and diseases are extensively dissected. In parallel, this review underscores the limitations of spore-forming probiotics, focusing on safety concerns, issues related to dose standardization and quality control, as well as potential off-target effects and risks in immunocompromised individuals.

## 1. Introduction

The gastrointestinal (GI) tract hosts a complex ecosystem composed of a diverse array of microorganisms, collectively referred to as the gut microbiota. The gut microbiota closely interacts with its host, regulating and supporting different functions, including the immune, hormonal, and nervous system through complex signaling pathways [1,2,3]. It also helps in maintaining gut homeostasis, influencing various physiological processes, such as digestion, nutrient absorption, and metabolism [4,5]. In contrast, the dysregulation of host–microbe interactions has been implicated in the pathogenesis of various diseases, including inflammatory bowel diseases (IBD), obesity, and metabolic syndrome [2]. Probiotics have garnered significant attention from the scientific and medical communities as they offer an effective and non-invasive restoration of this dysregulation, resulting in a myriad of potential health benefits [6].

Of the different species of probiotics consumed around the world for their health benefits, Gram-positive bacteria belonging to the genus *Bacillus* or related genera, such as *Alkalihalobacillus* and *Heyndrickxia*, are among the most extensively studied and are characterized by the unique ability to form endospores [7,8,9].

Endospores could be defined as dormant, resistant, and non-reproductive structures produced by certain bacteria belonging to the phylum *Bacillota* (formerly *Firmicutes*) in adverse conditions. When conditions become favorable for germination, spores can revert to the vegetative state and resume their metabolic activities.

Spores are highly resistant to harsh environmental conditions, including extreme temperatures, pH, and desiccation. This remarkable resistance can be attributed to their unique properties, such as a thick proteinaceous coat, high levels of calcium-dipicolinic acid (Ca^2+^-DPA) in the spore core, a low water content, and minimal metabolic activity [10,11]. Additionally, spore DNA is protected by its saturation with α/β-type small acid-soluble proteins (SASPs), which bind to DNA, altering its conformation and making it resistant to damage [10]. Such characteristics enable spores to survive in extreme environments, but also to persist in the host’s GI tract and exert their beneficial effects [12]. It was indeed observed that commercial probiotics containing members of the genus *Bacillus* and related genera, such as *Bacillus cereus*, *Alkalihalobacillus clausii* (formerly *Bacillus clausii*), and *Bacillus pumilus,* could persist in the mouse GI tract for up to 16 days [13]. Among these spore-forming species, *A. clausii* and *Heyndrickxia coagulans* (formerly *Bacillus coagulans*) represent the most largely commercialized probiotics worldwide. Various studies highlighted the distinguishing physiological properties of these microorganisms, including their tolerance to harsh GI environments, the enhancement of the gut barrier function, broad-spectrum antibiotic resistance, and their ability to synthesize vitamins [14,15,16,17,18].

Although spores are often included in commercial probiotic formulations, the importance of these forms of resistance has been under-appreciated from a clinical and microbiological perspective. In order to elucidate the therapeutic potential of spore-forming probiotics and optimize their clinical applicability, it is essential to comprehend their mechanisms of action. In view of the above, this review aims to shed more light on the distinctive attributes of spore-forming bacteria as probiotics, with a particular emphasis on the advantages conferred by their ability to form spores. Since an extensive review on *H. coagulans* has been recently published [19], herein we will particularly focus on the use of *A. clausii* as a probiotic, discussing its potential applications in health and disease.

## 2. Key Attributes of Spore-Forming Probiotics

### 2.1. Resistance to Extreme Conditions

The GI tract provides a challenging environment for bacterial survival due to partial or complete anoxia, low pH, bile salts, and an extremely high concentration of commensal microbes that compete for nutrients and the ecological niche [20].

**Figure 1:**
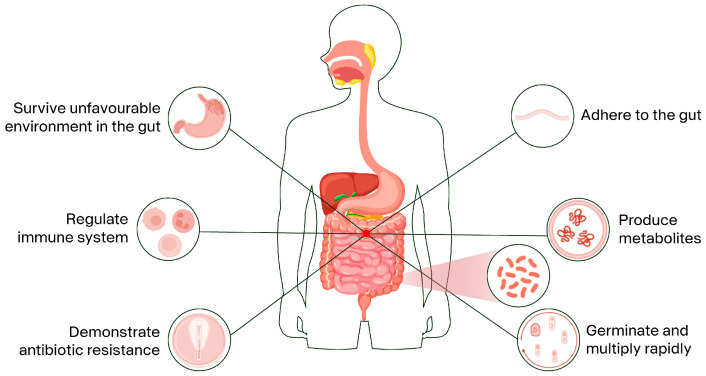
Mechanism of action of spore-forming probiotics.

The efficacy of orally administered probiotics depends on their ability to resist these conditions, colonize the gut, and exert functional benefits [15,21,22,23]. In this context, bacterial spores are known to remain inert and transit unrestrained through the gut (Figure 1) despite unfavorable conditions [20]. Spores of *A. clausii* were reported to survive in acidic environments, in the presence of bile and anoxic conditions [15,24].

Spore germination involves tightly regulated transcriptional changes followed by morphological cell changes [25]. The events typically include rehydration, the release of Ca^2+^-DPA and other core components, followed by the formation of metabolically active vegetative cells [26]. Once formed, the vegetative cells become less resistant to environmental stress. Despite this, they can adapt to specific environments at different stages of their life cycle. This adaptation is made possible through responses that adjust to the environment, which are controlled by complex molecular mechanisms.

The H^+^ homeostasis in *A. clausii* was shown to be maintained by the F0F1 ATP synthase complex that works by hydrolyzing ATP to pump protons (H^+^) from the cytoplasm [16,24]. Various proteins involved in the general stress adaptation by *A. clausii* ENTPro were identified by their annotation and Pfam domain search [16]. Notably, the universal stress protein UspA (PF00582) encoded by a gene present in the *A. clausii* ENTPro genome was found crucial for survival during cellular growth arrest and reprogramming the cell’s defence mechanisms during stress in other bacteria [27,28]. Molecular chaperones, such as GroES (PF00166) and GroEL, can contribute to the resistance of *A. clausii* against environmental stressors. In addition, the heat shock protein 33 (PF01430), the cold shock proteins CspA (PF00313), Clp proteases (PF00574), and HtpX and HrcA-like heat shock proteins could help in maintaining cellular growth and nucleic acid stability, while preventing the formation of inclusion bodies. The chaperone protein DnaJ (PF00226) and the nucleotide exchange factor GrpE (PF01025) can provide resistance to hyperosmotic stress and heat. Moreover, two copies of the methionine sulfoxide reductase A-encoding gene can confer resistance to oxidative stress [16].

### 2.2. Germination and Multiplication

Spore-forming probiotics demonstrate extreme stability and resistance to unfavorable conditions, attributes that facilitate their transit in the gut and survival at the low pH of the gastric barrier [12]. Furthermore, their stability and survival could be improved using various strategies and technologies, such as inducing sub-lethal stress, drying, encapsulation, and immobilization [26,27,28,29,30,31,32,33,34]. After spore germination, vegetative bacteria can rapidly multiply (Figure 1), allowing the quick and temporary colonization of the gut [35].

In vitro studies demonstrated that specific germinant receptors on the spore surface recognize signals such as amino acids, sugars, and bile salts, triggering spore germination [36]. In the case of *Bacillus subtilis* spores, micromolar concentrations of peptidoglycan-derived muropeptides act as potent germinants [37].

In vivo studies on *Bacillus* spores indicated that they may have similar life cycles in mammals and avian species, but with species- and strain-specific differences in the survival and persistence of the spores in the gut [20]. However, studies elucidating the mechanisms of spore germination in the human gut are scarce. Moreover, the mechanisms are considered different from that of other mammals, as the majority of studies have been conducted in vitro using artificial GI tract models [38,39,40,41].

In a study conducted by Shinde and co-authors [42], the spores of *H. coagulans* MTCC 5856 were shown to tolerate simulated conditions mimicking the digestion process. In addition, the *H. coagulans* strain contained in a mono-strain commercial probiotic product was found to persist in artificial gastrointestinal fluids for prolonged time [43]. Vecchione et al. [15] compared the in vitro behavior of various probiotic formulations commercialized in Italy and reported the remarkable survival of a probiotic mixture containing four *A. clausii* strains, as simulated gastric fluids. In the same study, an about 3-Log increase in the number of *A. clausii* cells was observed after 240 min of incubation in an artificial intestinal juice, thus highlighting the ability of *A. clausii* to multiply in intestinal-like conditions [15]. Furthermore, in a randomized, open-label, cross-over trial, Ghelardi et al. [44] investigated the GI fate of orally administered *A. clausii* using two commercial probiotic formulations containing spores of strains O/C, N/R, SIN, and T administered as a single dose to human volunteers. It was reported that the spores survived transit through the human gut and maintained considerable intestinal titer for up to 12 days after administration, and then underwent germination, outgrowth, and multiplication as vegetative forms. The strains also exhibited specific adaptation to the gut environment. In addition, an in vitro study by Mazzantini et al. [18] underscored the ability of *A. clausii* strains N/R and T and *H. coagulans* ATCC 7050 to survive in simulated intestinal fluids for as long as 8 h and the multiplication of vegetative cells of *A. clausii* O/C and SIN in these conditions. A systematic review of the literature by Morelli and Pellegrino [45] on the ability of viable probiotic strains to survive and reproduce in the gastrointestinal tract of healthy adults also highlighted a better survival of spore-forming probiotics in the GI tract. Lately, Ghelardi et al. [44] reported the survival in simulated gastrointestinal fluids of *A. clausii* spores contained in formulations, namely Enterogermina 4B, Enterogermina 6B, and Enterogermina Sporattiva. Also, the *A. clausii* SIN spores in Enterogermina Sporattiva were found to multiply in the simulated intestinal environment. In a recent in vitro study [46], *A. clausii* spores contained in Enterogermina 2B were shown to germinate in intestinal-like conditions following activation in a gastric juice. Additionally, *A. clausii* cells were shown to multiply in the intestinal fluid, increasing their number of about 3-Log [46].

### 2.3. Adhesion to the Gut

The adhesion of probiotic bacteria to the host gut is required for the competitive exclusion of gut pathogens, the release of antimicrobial substances, short-chain fatty acids (SCFAs), and organic acids, and the modulation of the host immune system [13,16,47,48]. The good adherence capacity of spore-forming probiotics (Figure 1) promotes the gut residence time, pathogen elimination, and host immune modulation [16]. The spore coat and exosporium were found to have a crucial role in spore adhesion [4]. Among the key proteins identified on the surface of the spores are mannose-specific lectins and spore-coat-associated proteins [49]. A Pfam analysis of the surface proteins of Gram-positive bacteria revealed the involvement of a mucus-binding protein with the ‘Gram_pos_anchor’ Pfam domain (PF00746) at the *C-*terminus, a collagen-binding protein with an LPXTG motif at the *C-*terminus, and a fibronectin-binding protein in facilitating the adhesion of a probiotic bacterium to the intestinal mucosal layer and further interactions [16,50]. In addition, adhesion is determined by many factors such as cell wall hydrophobicity, the adhesion potential, and auto-aggregation [51,52,53]. With few exceptions, spores exhibit higher adhesion compared to vegetative cells [49,54]. This could be attributed to their higher hydrophobicity and lower energy barrier, compared to vegetative cells [55]. In fact, the spores of *H. coagulans* MTCC 5856 were shown to strongly adhere to human colonic cell lines [42]. Vegetative cells of *H. coagulans* ATCC 7050 and *A. clausii* O/C, N/R, SIN, and T and spores of *A. clausii* were found to adhere to gastrointestinal mucins, thus highlighting their potential ability to colonize the mucus layer of the gut [18,46]. This behavior was supposed to be attributed to the presence of mucus-binding proteins and type-IV filamentous adhesions on the cell surface [16,18,56].

### 2.4. Production of Bioactive Compounds

Spore-forming probiotics produce various active metabolites (Figure 1), including SCFAs, such as acetate, propionate, and butyrate, as a result of dietary carbohydrate and protein digestion [57,58]. SCFAs are volatile fatty acids produced by the gut microbiota in the large bowel as fermentation products from food components that are unabsorbed/undigested in the small intestine [58]. SCFAs have distinct physiological effects. They contribute to shaping the gut environment, influence the physiology of the colon, and can be used as energy sources by host cells and by the intestinal microbiota [58]. They also participate in different host-signaling mechanisms [58]. In addition, SCFAs promote gut microbiota-mediated alterations in the gut motility through the maintenance of colonic serotonin homeostasis and participate in different host-signaling mechanisms [58,59]. SCFAs can serve as energy sources for host cells and for the gut microbiota to conduct varied functions in the host intestine; thus, their amount and relative abundance may be considered as biomarkers of a healthy status. Reduced levels of acetic, propionic, and butyric acids in the gut often coincide with health issues, suggesting that supplementing with SCFA-producing microbes could alleviate related symptoms. Calvigioni et al. [60] demonstrated for the first time that *A. clausii* strains N/R, O/C, SIN, and T can produce acetic, propionic, and butyric acids in vitro, thus suggesting that they could produce these molecules also in vivo. Interestingly, the strain T resulted to be the highest producer of acetic acid (602.00 ± 54.15 ng/mL) and was found to secrete about two-fold more propionic acid than *A. clausii* NR and SIN. In contrast, no differences in the amount of secreted butyric acid were evidenced between the four *A. clausii* strains. The production/secretion of these SCFAs in vitro, particularly acetic acid, was recently demonstrated for a formulation containing a spore mixture of *A. clausii* N/R, O/C, SIN, and T [46]. In the study from Calvigioni and colleagues, the production of acetic acid (~300 ng/mL), but not of propionic and butyric acids, was evidenced also for *H. coagulans* ATCC 7050 [60]. Interesting, the effect of the *A. clausii* strains on SCFA production was also highlighted in a model of the GI tract of patients treated with proton pump inhibitors, with the increased production of butyrate and decreased production of acetate, thus suggesting an effect on the overall production of these molecules [5,61].

The administration of probiotics producing B-group vitamins is advantageous for human health, since these vitamins play a pivotal role in the metabolism of carbohydrates, proteins, and fatty acids, in the prevention of oxidative stress, and in the maintenance of neuronal functionality [62]. Mazzantini et al. [18] demonstrated in vitro the synthesis of vitamins, particularly vitamin B2 (riboflavin), by the vegetative *A. clausii* strains O/C, N/R, SIN, and T, as well as by *H. coagulans* ATCC 7050. In particular, the amount of vitamin B2 produced by *H. coagulans* ATCC 7050 was found to be more than 10-fold higher than that produced by the *A. clausii* strains [18]. Riboflavin produced by the *A. clausii* strains was previously found to support the growth of a riboflavin-deficient mutant of *B. cereus* on riboflavin-depleted media [14]. In addition, the secretion of vitamins B2 (22.86 ± 1.61 ng/mL), B8 (3.93 ± 0.01 ng/mL), B9 (2.01 ± 0.12 ng/mL), and B12 (1.17 ± 0.64 ng/mL) was recently demonstrated in vitro for a formulation containing spores of the same *A. clausii* strains [46].

Over recent decades, numerous studies have suggested that probiotics play a crucial role in preventing oxidative stress and in various diseases associated with reactive oxygen species (ROS) by producing antioxidants. Specifically, *Bacillus* and related genera are known to produce enzymes like catalase (CAT) and superoxide dismutase (SOD), which help the host in counteracting oxidative stress [63,64]. Lippolis and coauthors identified CAT in the secretomes of *A. clausii* O/C, N/R, SIN, and T, with strains O/C and N/R secreting higher levels of the proteins than the other strains [65]. In the same study, all the *A. clausii* strains were found to secrete SOD and the strain O/C resulted to be the highest producer. Thereafter, CAT and SOD activities were detected in both cell lysates and culture supernatants obtained from *H. coagulans* ATCC 7050, *A. clausii* O/C, N/R, SIN, and T, and from the formulation containing a spore mixture of these *A. clausii* strains [18,46]. An in vitro study hypothesized that the administration of these probiotic strains could potentially reduce or alleviate ROS accumulation in vivo, thus preventing oxidative stress [18].

Spore-forming bacteria are known to produce a variety of bioactive enzymes that promote food digestion and nutrients adsorption. These include proteases, lipases, and amylases, which digest proteins, lipids, and carbohydrates, respectively. Other relevant enzymes are α- and β-galactosidases, which hydrolyze complex galactooligosaccharides and lactose, respectively, and pectinases responsible for pectin digestion. A recent review of Nicolas and Ma [66] collected evidence on the ability of some probiotic strains of *H. coagulans* (e.g., KM-1, MA-13, NL01, RCS3, and GBI−30 6086) to produce α- and β-galactosidases, amylases, alkaline proteases, lipases, and/or pectinases in vitro. Furthermore, the production of β-galactosidase was demonstrated in vitro for *H. coagulans* ATCC 7050, *A. clausii* (i.e., O/C, N/R, SIN, and T), and for a formulation containing spores of these *A. clausii* strains [18,46]. Interestingly, *H. coagulans* ATCC 7050 was found to produce two-fold more β-galactosidase than *A. clausii* N/R, O/C, and T, and three-fold more of the enzyme than *A. clausii* SIN [18]. Since this enzyme is responsible for the degradation of lactose, the ability of these microbes to produce β-galactosidase have been hypothesized to contribute in ameliorating the symptomatology associated with lactose intolerance [18].

The production of alkaline proteases was demonstrated in vitro for *A. clausii* I-52 and UBBC07 [67,68,69]. In addition, a comparative proteomic analysis of *A. clausii* O/C, N/R, SIN, and T identified an alkaline protease (AprE) and an aminopeptidase (AmpS) in the extracellular proteomes of all strains. Interestingly, stains O/C and T resulted in being able to secrete higher amounts of AprE compared to strains N/R and SIN [65]. Interestingly, Lin and coauthors [70] evidenced the ability of *B. subtilis* ubsp. *natto* NTU-18 to produce a nattokinase able to interfere with the transfer of vancomycin resistance in *Enterococcus faecalis*.

### 2.5. Production of Antimicrobial Compounds

*Bacillus* spp. produce various antimicrobial compounds that aid in the competitive exclusion of pathogenic microbes in the gut [71,72,73]. These compounds include bacteriocins and bacteriocin-like inhibitory substances such as subtilin and coagulin, or lipopeptides, such as surfactin, iturins, bacillomycins, and mycosubtilins. Moreover, *Bacillus* spp. are well known producers of lantibiotics, post-translationally modified antimicrobial peptides that are active against Gram-positive bacteria and a few Gram-negative bacteria including pathogens and antibiotic-resistant bacteria [71,74,75,76,77,78].

*H. coagulans* strains can secrete a bacteriocin, a well-known antibacterial substance, as well as other antimicrobial substances, such as lactic acid and its derivatives, which may be produced and act in the human gut [79,80,81,82].

In a preclinical study by Paparo et al. [83], the protective mechanisms of *A. clausii* against the rotavirus infection have been elucidated, providing insights into the mechanistic basis underlying the clinical efficacy of *A. clausii* in pediatric viral acute gastroenteritis. Using the human pediatric enterocyte model, it was demonstrated that vegetative cells of *A. clausii* strains O/C, N/R, SIN, and T induce the synthesis of antimicrobial peptides, human beta defensin 2, and cathelicidin, that aid in fighting infections and rescuing cell proliferation, which is impaired by rotavirus. Additionally, *A. clausii* strains were reported to inhibit ROS production by rotavirus and suppress the release of pro-inflammatory cytokines, such as interleukin (IL)-8 and interferon (IFN)-β, and genes associated with the Toll-like receptor (TLR)-3 pathway [83].

The vegetative cells of *A. clausii* produce antimicrobial peptides with inhibitory effects against a broad spectrum of bacteria, including *Salmonella typhimurium*, *Escherichia coli*, *Shigella flexneri*, *Staphylococcus aureus*, *Listeria monocytogenes*, and *Enterococcus faecalis*. Urdaci et al. [84] reported that the release of these antimicrobial substances occurs during a stationary growth phase and coincides with sporulation. Specifically, two strains of *A. clausii*, UBBC07 and O/C, were found to produce in vitro a peptide called clausin that demonstrates antimicrobial activity primarily against Gram-positive bacteria. In addition, clausin also mitigates the cytotoxic effects on Caco2 cells induced by *Clostridioides difficile* [77,85]. It also targets lipid intermediates involved in bacterial peptidoglycan synthesis, providing further insights into its mechanism of action against bacterial pathogens [86].

### 2.6. Antibiotic Resistance

The presence of antibiotic resistance genes (ARGs) on mobile genetic elements (i.e., plasmids, transposons, and phages) has been highlighted as a critical safety concern [87,88]. In fact, these genes can be transmitted to other microbes by a horizontal gene transfer, thus contributing to the spreading of antibiotic resistance. In contrast, the existence of non-transmissible AGRs stably residing in bacterial chromosomes could be considered an added value since some probiotics are administered in concomitance with antibiotics [16,89]. Several ARGs that enable probiotics’ survival and persistence during antibiotic treatment have been identified within the genomes of *Bacillus* spp. For example, *cfr*-like genes that encode ribosome methyltransferases have been identified in several *Bacillus* spp. These genes determine the ability of *Bacillus* spp. to develop resistance against multiple antibiotic classes including phenicols, oxazolidinones, lincosamides, pleuromutilins, and streptogramin A [90]. Probiotic *A. clausii* strains harbor specific antibiotic defence genes such as an aminoglycoside resistance gene (*aadD2*), a chloramphenicol acetyltransferase gene (*cat(Bcl*)), and a β-lactamase gene (*bla*_BCL-1_) that contribute to their ability to withstand antibiotic pressure and maintain their probiotic function in the gut environment [91,92,93].

Multidrug resistance proteins, universal stress proteins, the EmrB multidrug efflux system, tetracycline resistance proteins, and different classes of beta-lactamases are present in various *Bacillus* spp., conferring resistance against common antibiotics (Figure 1). However, vancomycin resistance was only identified in *Bacillus toyonensis* and chloramphenicol resistance in all included bacteria except for *H. coagulans*, highlighting the diversity of antibiotic resistance mechanisms within the genus *Bacillus* and related genera [16]. In addition, it was reported that the antibiotic sensitivity patterns of four *A. clausii* strains, O/C, SIN, N/R, and T, differ from that of the wild *A. clausii* strain DSM8716. Although the strains show similarities, such as resistance to macrolides, beta-lactams, and aminoglycosides, *A. clausii* DSM8716 is sensitive to chloramphenicol, rifampicin, streptomycin, and tetracycline, indicating that the probiotic strains O/C, SIN, N/R, and T have acquired resistance to these compounds through selective mutagenesis [94]. Furthermore, the analysis of mutations involved in the resistance to streptomycin and rifampicin revealed the K101R mutation in the *rpsL* gene, associated with streptomycin resistance in the SIN strain, and the S488F mutation in the *rpoB* gene, associated with rifampicin resistance in the N/R strain [85].

The analysis of the composite genome of *A. clausii* ENTPro confirmed the presence of chromosomic genes encoding chloramphenicol acetyltransferase, tetracycline resistance domains, two proteins with the streptomycin adenylyltransferase domain (PF04439), six proteins with the aminoglycoside phosphotransferase (PF01909) domain, and one protein with the kanamycin nucleotidyltransferase (PF07827) domain imparting resistance to streptomycin [16]. The identification of specific protein domains associated with antibiotic resistance in *A. clausii* highlights the diverse repertoire of molecular mechanisms underlying its ability to resist certain antibiotics as well as the differences between bacteria of the same species [16].

### 2.7. Regulation of the Immune System

The intricate interplay between the gut microbiota and the mucosal immune system profoundly influences the maintenance of the gut immune system and metabolic homeostasis [95]. Probiotics exert their immunomodulatory effects through direct interactions with the intestinal epithelium, particularly in the small intestine, where the commensal microbiota are less abundant [96]. Additionally, through various effector molecules, including bacterial cell wall components, such as peptidoglycan and lipoteichoic acid, as well as specific proteins, probiotics moderate host immune responses by modulating receptor signaling cascades involved in the immune system regulation [97,98]. Beyond the conventional immune functions, emerging evidence highlights the role of the gut microbiota and their components in regulating energy, glucose, and lipid metabolism, demonstrating a link between them and host metabolism [99,100,101]. Thus, manipulating the gut microbiota using probiotics offers promising avenues for modulating host metabolism and potentially managing metabolic disorders [102,103].

The immunomodulatory effect of spore-forming probiotics can be attributed to cell wall components, surface-associated proteins, glycoproteins, and secreted factors. An in vitro evaluation of the immunomodulatory properties of whole vegetative cells of *A. clausii* on Swiss and C57 Bl/6j murine cells revealed the induction of nitric oxide synthetase activity, IFN-γ production, and CD4+ T-cell proliferation [84]. The cell wall fraction of *H. coagulans* JBI-YZ6.3 was shown to increase the level of expression of CD25 and CD69 activator markers on NK, T, and dendritic cells, as well as on monocytes [104]. Interestingly, CD69 expression on monocytes has been linked to the activation of the 5-lipoxygenase pathway, which results in the production of leukotrienes responsible for immune cells’ recruitment [105]. In addition, the same fraction was found to increase the production of IFN-γ and some pro-inflammatory cytokines, as well as of the stem cell-mobilizing growth factor (G-CSF), thus enhancing post-injury and post-inflammation repair processes [104]. The immunomodulatory activity exerted by the cell wall fraction reasonably involves different bacterial glycans and host pattern recognition receptors. In fact, as described for other Gram-positive probiotics, peptidoglycan and its components muramyl dipeptide and N-acetylglucosamine can be recognized by the Toll-Like Receptor (TLR) 2, leading to the activation of the Nuclear Factor kappa-light-chain-enhancer of activated B cells (NF-κB) and the Mitogen-Activated Protein Kinase (MAPK) pathways [106,107,108]. Furthermore, the D-alanine in the cell wall can amplify TLR2 signaling, directly impacting cytokine production [109]. Additionally, muramyl dipeptides in the cell wall can activate the nucleotide-binding oligomerization domain 2 (NOD2) receptors, which stimulates the activation of NF-κB and MAPK pathways through the recruitment of the receptor-interacting protein kinase 2 (RIP2) [107,108]. An elegant in vitro study from Zhang and coworkers [110] clarified the possible mechanism of action of the cell wall lipoteichoic acid of *H. coagulans* HOM5301 in the activation of RAW 264.7 macrophages. In particular, lipoteichoic acid of this strain appeared able to stimulate TLR2 and induce the activation of MAPK and NF-κB pathways by recruiting the Myeloid Differentiation Primary-Response Protein 88 (MyD88) and the Interleukin-1 Receptor-Associated Kinases (IRAK), which in turn induce the expression of pro-inflammatory cytokines and chemokines [110].

To the best of our knowledge, no reports evaluating the specific immunomodulatory properties of isolated surface-associated proteins of spore-forming probiotics are available. Nevertheless, these molecules can also play a key role in immunomodulation exerted by these microbes. For instance, the interaction between bacterial s-layer proteins and the dendritic cell-specific ICAM-3-grabbing non-integrin (DC-SIGN), SIGN3, Decitin-1, and macrophage-inducible C-type lectin can help in the immune response modulation and protection of the gut barrier integrity, as described for other Gram-positive probiotics [107]. Furthermore, as observed in *Limosilactobacillus reuteri*, mucus-binding proteins can enhance immunomodulation by the binding of C-type lectin receptors, thus inducing a Th1-polarized immune response [111]. Glycoproteins constituting surface appendages (pili and flagella) can also be involved in the stimulation of the immune response induced by spore-forming bacteria, being recognized by TLR2 receptors [107].

Regarding secreted factors, *A. clausii* supernatants were observed to protect intestinal cells from cytotoxicity induced by *C. difficile* and *B. cereus* through a serine protease, which rescues cell viability and mitochondrial activity, proving its clinical relevance in the prophylaxis of *C. difficile*-associated diarrhea [78].

Zhu et al. [103] highlighted that *Bacillus*-derived metabolites, such as organic acids and exoenzymes, play a crucial role in modulating the host immune response (Figure 1). These metabolites enhance the host’s immune regulation by promoting the production of anti-inflammatory cytokines and inhibiting the growth of pathogenic bacteria. For instance, SCFAs can activate G-protein-coupled receptors (GPCRs), improving the production of anti-inflammatory factors by T-cells and reducing pro-inflammatory molecules [94]. Furthermore, pyruvate produced by bacilli can induce the GPCR-mediated dendrite protrusion of C-X3-C motif chemokine receptor 1+ cells, triggering an immune response. In particular, activated GPCRs modulate the host immune response through different signaling cascades, including the MAPK, the signal transducer and activator of transcription 3 (STAT3), and the mammalian target of rapamycin (mTOR) pathways [112]. These interactions underscore the potential of spore-forming probiotics in maintaining immune homeostasis and protecting against enteric infections. Cell wall components and metabolites from *H. coagulans* PTA-6086 have been shown to increase the activity of different immune cells [113,114].

*A. clausii* strains or their supernatants also improved the gut barrier integrity by reducing necrotic or apoptotic enterocytes, increasing mucin production, and enhancing the synthesis of tight junction proteins in an in vitro model of retrovirus infection [76]. *A. clausii* CSI08 cells and their supernatants were shown to attenuate the expression of pro-inflammatory cytokines by down-regulating the NF-κB transcription factor [115].

An in vitro study from Vicente-Gil and coauthors [116] investigated the immunomodulatory effects of extracellular vesicles produced by probiotic *B. subtilis* ABP1 on the intestinal epithelial cell line RTgutGC and splenic leukocytes. In both cell lines, vesicles were found to increase the expression of genes encoding some pro-inflammatory cytokines (i.e., IL-1β and IL-8) and antimicrobial peptides (i.e., hepcidin and cathelicidin 2). Furthermore, they stimulated the expression of genes correlated with B-cell differentiation and the production of MHC-II and IgM in splenic leukocytes [116]. Interestingly, the authors evidenced markedly different effects when vesicles obtained from *Bacillus megaterium* were used, thus suggesting a species- or even strain-dependent immunostimulatory activity [116].

Some in vivo studies also highlighted the immunomodulatory properties of spore-forming probiotics. Bomko and co-workers revealed that *H. coagulans* is able to normalize the number of splenic lymphocytes, macrophages, and T-lymphocytes and their functional activity to promote the host immune system when administered to mice with colitis [117]. Similarly, the administration of *A. clausii* (O/C, SIN, N/R, T) to mice alleviates the symptoms of colitis by modulating the gut microbiota [118]. Zhao and coworkers [119] investigated the in vivo immunomodulatory properties of *H. coagulans* MZY531 whole cells administered to cyclophosphamide-induced immunosuppressed mice. The authors evidenced the downregulation of TLR4/MyD88/NF-κB pathways and the upregulation of anti-inflammatory cytokines (INF-γ, IL-2, IL-4, and IL-10) [119].

*A. clausii* demonstrated therapeutic potential, indicating its clinical applicability in various inflammatory conditions, as evidenced by the following studies. In a mouse model of ovalbumin-induced asthma, *A. clausii* administration reduced eosinophils, neutrophils, and lymphocytes, while also attenuating airway epithelium thickening and decreasing IL-4 and IL-5 levels [120]. Additionally, *A. clausii* MTCC-8326 induced a controlled inflammatory response in murine macrophages, balancing pro-inflammatory and anti-inflammatory cytokine expression [121]. In mice with schistosomiasis, *A. clausii* increased the levels of the anti-inflammatory cytokine IL-10, Treg, and Th17 cells and reduced the levels of the pro-inflammatory cytokines IFN-γ, tumor necrosis factor (TNF)-α, and IL-6, resulting in a reduction in inflammation [122].

## 3. *A. clausii* in the Treatment Landscape of Various Diseases

Many probiotic formulations containing *A. clausii* spores are commercialized around the world [18,43,123,124,125,126]. Most of these formulations contain only one *A. clausii* strain (i.e., UBBC-07, 088AE, SIN), while others contain more than one strain or a combination of *A. clausii* and other bacteria. In many studies, a formulation consisting of spores of the *A. clausii* strains O/C, N/R, SIN, and T (Enterogermina) has been used. This product is licensed and marketed as a prophylactic medicine in more than 50 countries around the world [9,73] for its demonstrated tolerability and efficacy in humans over several decades [44,94,127,128].

Despite their extensive use and proven efficacy, *A. clausii* probiotics have been relatively less reported and disseminated in the literature compared to probiotics belonging to other genera such as *Lactobacillus* and *Bifidobacterium* [7]. Several clinical studies proved the efficacy of *A. clausii* spores in the management of various health disorders (Table 1).

## 4. Safety, Regulatory-Related Considerations, and Limitation of Spore-Forming Probiotics

Spore-forming bacteria are commonly included in a plethora of commercial formulations that are sold as foods, food supplements, or drugs/live biotherapeutic products (LBPs), depending on the intended use [149,150].

Regarding safety, bacteria used as probiotics in foods and food supplements should have a long history of safe use in humans and should possess the Generally Recognized As Safe (GRAS) or the Qualified Presumption of Safety (QPS) status [87,151,152]. In contrast, microbes contained in drugs/LBPs undergo meticulous safety assessments by performing in vitro and in vivo studies, as well as clinical trials in humans [88].

A limited number of cases of bacteremia and sepsis in immunocompromised patients or individuals with underlying comorbidities, due to the consumption of *A. clausii* probiotics, have been published [153,154]. The development of bacteremia and sepsis following the administration of products containing *A. clausii* has also been reported in two patients hospitalized in the intensive care unit [155], in an infant with short bowel syndrome [156], in an 87-year-old man [157], and in a pediatric patient affected by a rare autoinflammatory disease [158]. Furthermore, cases of fatal septic shock in a 4-month-old infant with congenital heart disease [159] and in an infant with malnutrition, acute diarrhea, and moderate-to-severe dehydration [160] have been reported. In light of this evidence, physicians should exercise caution while considering the prolonged administration of spore-forming preparations to immunocompromised individuals and subjects with underlying comorbidities.

Spore-forming bacteria, particularly those constituting drugs/LBPs, should adhere to rigorous standards related to potency, identity, and purity [161]. Regarding potency, commercial products should contain the number of living microbes labeled by manufacturers, since this amount is crucial for providing the desired beneficial effect to consumers [162]. Compared to other probiotics, spore-forming microbes have the advantage that spores can survive unaffected during the industrial production process and for the entire product shelf-life. Nevertheless, the use of appropriate methods for enumerating spores at the time of manufacturing is important to guarantee potency [161]. In fact, a different number of spores than that labeled was evidenced in different studies investigating the quality of some *Bacillus*-containing products [43,123,124,125,126].

The United States Food and Drug Administration (FDA) and the European Medicines Agency (EMA) require that microbes included in drugs/LBPs are identified at the strain level and genotypically and phenotypically characterized [88]. The identification of microbes used in LBPs should be accurately performed, possibly using two different methods, as recommended by the FDA and EMA, to guarantee the strain identity and product purity [88]. Regarding probiotics included in foods and food supplements, the Food and Agriculture Organization of The United Nations (FAO) and the World Health Organization (WHO) defined the methods to be applied for their correct identification [151]. The use of inappropriate identification methods combined with flaws in manufacturer quality controls could result in the inclusion of different *Bacillus* strains, contaminant microorganisms, and even potential pathogens in commercial products, as evidenced in some studies [123,124,125,126].

The characterization of spore-forming strains is aimed at excluding the presence of virulence factors (e.g., toxins) that can constitute a potential risk for consumers. Although the presence of chromosomal non-transmissible ARGs is tolerated, probiotic spore-formers must not possess ARGs localized on mobile genetic elements, since these genes can be transmitted to gut commensals or pathogenic microbes through a horizontal gene transfer [87,88,151]. Although the strains analyzed in this review only possess chromosomal and non-transmissible ARGs, spore-forming microbes should always be screened to exclude the presence of transmissible ARGs before their inclusion in foods, food supplements, or drugs/LBPs.

As highlighted in this review, some of the beneficial properties exerted by spore-forming bacteria (e.g., multiplication in simulated intestinal fluid, the production of SCFAs) appeared to be species- or even strain-dependent. For this reason, the choice of the right product to administer should take into account its microbial composition and should be personalized based on the specific requirements of each individual. In parallel, it is also important to mention that some attributes of spore-forming bacteria (e.g., mucin adhesion, spore germination, and vegetative cell multiplication in a gut-like environment) could be unwanted in immunocompromised people. In fact, these properties can enhance the persistence of bacilli in the human gut, thus increasing the risk of off-target effects ranging from digestive discomfort to blood translocation and systemic infections [88]. Compared to other probiotics (e.g., lactobacilli, bifidobacteria), a limited number of papers investigating the beneficial properties of spore-forming bacteria are available, thus leading the knowledge on the potential of these microbes still being inadequate. We believe that more in vitro and in vivo studies should be performed to dissect almost all the attributes of these microbes, thus expanding the range of beneficial effects they could provide for human health. It is also important to remark that most of the properties evidenced in this review come from in vitro studies or in vivo investigations conducted on animal models, thus limiting their translational value. Therefore, new clinical studies should be performed for validating the beneficial attributes of spore-forming bacteria also in humans.

## 5. Conclusions

Spore-forming probiotics, such as *H. coagulans* and *A. clausii*, exhibit unique properties and mechanisms of action that distinguish them from non-spore-forming probiotics, providing alternative viable solutions for enhancing digestive health and overall well-being. The ability to form spores enables these microbes to withstand harsh environmental conditions, such as high temperatures and an acidic pH, thereby enhancing the stability and shelf life. Spore-forming bacteria that adhere to gastrointestinal mucins and *A. clausii* can multiply in gut-like conditions, thus emphasizing the ability to persist in the human intestine for prolonged times. In addition, spore-forming probiotics display promising immunomodulatory properties and appear able to produce antimicrobial molecules and bioactive compounds (i.e., SCFAs, B-group vitamins, and enzymes) that can improve human health. Despite their promise, it is essential to approach their use with a balanced perspective. As research in this field continues to advance, harnessing their potential holds prospects for improving human health and combating a range of human disorders. In addition to their beneficial effects, some limitations to the use of spore-forming probiotics have been evidenced (paragraph 4) and should be taken into account when these microbes are considered for human consumption.

## Figures and Tables

**Table 1:** Clinical studies reporting the efficacy of *A. clausii* in various disorders.

Indication	Study Design	Population	*A. clausii* Strain or Formulation	Dosing	Reference
Acute diarrhea	Phase III, randomized, double-blind, placebo-controlled	Children	O/C, N/R, SIN, T	2 × 10^9^ spores twice a day for 5 days	[129]
	Double-blind, placebo-controlled	Children	UBBC-07	2 × 10^9^ spores twice a day for 5 days	[130]
	Prospective, randomized, single-blind, controlled	Children	O/C, N/R, SIN, T	2 × 10^9^ spores twice a day for 5 days	[131]
	Prospective, randomized, single-blind, controlled	Children	O/C, N/R, SIN, T	10^9^ spores twice a day for 5 days	[132]
	Prospective, randomized, controlled, open labeled	Children	19T, 21C, 28S, 30R	2 × 10^9^ spores twice a day for 3 days	[133]
	Prospective, randomized, double-blind, placebo-controlled	Children, adolescents, adults	088AE	Children: 2 × 10^9^ CFU twice a day for 7 days; Adolescents and adults: 2 × 10^9^ CFU thrice a day for 7 days	[134]
Persistent diarrhea	Randomized, double-blind, controlled	Children	LiveSpo Clausy strain	4 × 10^9^ spores thrice a day for 3 days and twice a day for 7 days	[135]
Community-acquired diarrhea	Prospective, open-label, observational	Children	O/C, N/R, SIN T	2 or 4 × 10^9^ spores/day for 5–7 days	[136]
*Helicobacter pylori* infection	Prospective, randomized, double-blind, placebo-controlled	Adults	O/C, N/R, SIN, T	2 × 10^9^ CFU thrice a day for 14 days	[137]
	Phase IIIB, randomized, double-blind, placebo-controlled	Adults	O/C, N/R, SIN, T	2 × 10^9^ CFU thrice a day for 14 days	[138]
Rotavirus infection	Prospective, controlled	Children	Data not available	Data not available	[139]
	Prospective, controlled	Children	Data not available	Data not available	[140]
Irritable bowel syndrome	Randomized, double-blind, placebo-controlled	Children and adolescents	O/C, N/R, SIN, T	4 × 10^9^ CFU/day for 6 weeks	[141]
Recurrent aphthous ulcer and oral candidiasis	Randomized controlled	Adults	Not defined strain	Topical application twice a day for 1 week	[142]
Undiagnosed GI discomfort	Prospective, double-blind, placebo-controlled	Adults	*H. coagulans* SNZ 1969, *A. clausii* SNZ 1971, and *B. subtilis* SNZ 1972	2 × 10^9^ CFU once a day for 30 days	[143]
Endotoxemia	Randomized, double-blind, placebo-controlled	Adults	*B. indicus* HU36, *B. subtilis* HU58, *H. coagulans*, *B. licheniformis*, *A. clausii*	for 30 days	[144]
Necrotizing enterocolitis and late-onset sepsis	Randomized, double-blind, placebo-controlled	Infants	O/C, N/R, SIN, T	2 × 10^9^ spores per day	[145]
Respiratory infections	Randomized, single-blind	Children	O/C, N/R, SIN, T	2 × 10^9^ spores twice daily for 90 days	[146]
	Single-blind, pilot study	Children	O/C, N/R, SIN, T	2 × 10^9^ spores twice daily for 4 weeks	[147]
Respiratory, genitourinary, or skin and soft tissue bacterial infections	Pooled analysis of three controlled trials	Children	O/C, N/R, SIN, T	2 × 10^9^ spores per day ≥ 5 days (one study), 20 days (one study)	[148]

CFU, colony-forming units; GI, gastrointestinal.

## Data Availability

No new data were created or analyzed in this study. Data Sharing is not applicable to this article.
